# Comparative Study of Blood Neopterin and Biopterins in Patients with COVID-19 and Secondary Bacterial Infection

**DOI:** 10.3390/jcm13154365

**Published:** 2024-07-26

**Authors:** Tomohiro Eguchi, Shuhei Niiyama, Chinatsu Kamikokuryo, Yutaro Madokoro, Kenshin Shimono, Satoshi Hara, Hiroshi Ichinose, Yasuyuki Kakihana

**Affiliations:** 1 Department of Emergency and Intensive Care Medicine, Kagoshima University Graduate School of Medical and Dental Sciences, Kagoshima 890-8544, Japan; tomohiro19860704@gmail.com (T.E.);; 2 Emergency and Critical Care Center, Kagoshima City Hospital, Kagoshima 890-8760, Japan; 3 Department of Life Science Research for Emergency Intensive Care, Kagoshima University Graduate School of Medical and Dental Sciences, Kagoshima 890-8544, Japan; 4 School of Life Science and Technology, Tokyo Institute of Technology, Yokohama 226-8501, Japan

**Keywords:** neopterin, biopterin, COVID-19, secondary bacterial infection

## Abstract

**Background/Objectives**: As COVID-19 can be severe, early predictive markers of both severity and onset of secondary bacterial infections are needed. This study first examined changes over time in the levels of plasma neopterin (NP) and biopterins (BPs), among others, in patients with COVID-19 and then in those with secondary bacterial infection complications. **Methods**: Fifty-two patients with COVID-19 admitted to two tertiary care centers were included. They were divided into a severe group (intubated + mechanical ventilation) (*n* = 10) and a moderate group (non-intubated + oxygen administration) (*n* = 42), and changes over time in plasma NP, plasma BPs, IFN-γ, lymphocyte count, CRP, and IL-6 were investigated. Four of the patients in the severe group (*n* = 10) developed secondary bacterial infections during treatment. Plasma NP and plasma BPs of patients with bacterial sepsis (no viral infection) (*n* = 25) were also examined. **Results**: The plasma NP, IL-6, CRP, and SOFA levels were significantly higher in the severe group, while the IFN-γ level and lymphocyte count were significantly lower. The higher plasma NP in the severe group persisted only up to 1 week after symptom onset. The plasma BPs were higher in complications of bacterial infection. **Conclusions**: The timing of sample collection is important for assessing severity through plasma NP, while plasma BPs may be a useful diagnostic tool for identifying the development of secondary bacterial infection in patients with COVID-19. Further investigation is needed to clarify the mechanism by which NP and BPs, which are involved in the same biosynthetic pathway, are differentially activated depending on the type of pathogen.

## 1. Introduction

The novel coronavirus disease 2019 (COVID-19) caused a pandemic worldwide; as of July 2023, 768 million people had been infected, and 6.95 million had died [1]. This novel SARS-CoV-2 virus causes only mild common cold symptoms, such as fever and cough, in most infected individuals; however, comorbidities such as old age [2], hypertension [3], diabetes [4], and chronic obstructive pulmonary disease [5] are considered risk factors for severe disease. Furthermore, complications regarding secondary bacterial infections in patients with COVID-19 infection have been observed in 8.4% of hospitalized patients and 39.9% of ICU patients [6]. The mortality rate of COVID-19 cases complicated by secondary bacterial infections has been reported to be 42.9% for early-onset infections (<48 h after admission) and 66% for late-onset infections (between 2 and 14 after admission) [7].

In viral infection, infected cells produce type I interferons (interferon-alpha [IFN-α] and interferon-beta [IFN-β]), which inhibit viral proliferation. Type I interferon induces the activation of NK cells, which in turn produce type II interferon (interferon-gamma [IFN-γ]) and activate macrophages as they eliminate the virus. The recognition of invading pathogen antigens by CD4+ T cells activates their differentiation into type 1 helper T lymphocytes (Th1s) and type 2 helper T lymphocytes (Th2s). Th1 cells release IFN-γ and various cytokines, further activating macrophages. Activated CD8+ T cells differentiate into cytotoxic T lymphocytes, which eliminate infected cells. This form of immunity is commonly referred to as cellular immunity. Severe lymphocyte depletion and type I interferon suppression have been reported in SARS-CoV-2 infection [8]. However, the excessive activation of T cells has also been reported to be associated with worse clinical outcomes [9], suggesting that a breakdown of the immune system worsens the disease.

**Figure 1:**
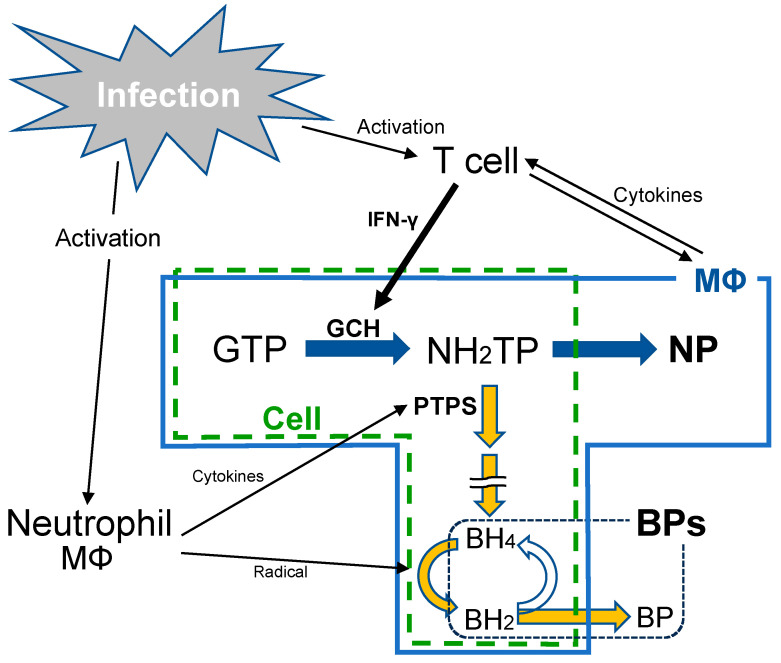
Schema of infection and neopterin (NP) and total biopterin (BP) biosynthetic pathway. NP is produced by activated monocytes and macrophages (MΦ) after the conversion of guanosine triphosphate (GTP) to 7,8-dihydroneopterin triphosphate (NH2TP) via GTP cyclohydrolase I (GCH). GCH mRNA expression is induced by IFN-γ released from activated Th1s and other cells. Tetrahydrobiopterin (BH4) is produced from NH2TP via several enzymatic reactions, including 6-pyruvoyl tetrahydropterin synthase (PTPS). BH4 is then oxidized to dihydrobiopterin (BH2), and the final oxidation product is biopterin (BP). IFN-γ, interferon-γ; Th1, helper T lymphocyte.

In vivo, biopterins exist mostly as tetrahydrobiopterin (BH4); however, some exist in oxide forms, dihydrobiopterin (BH2) and biopterin (BP). The most stable of these compounds is BP in its final oxide. Conversely, neopterin (NP) serves as an intermediate metabolite of BH4 and is an established biomarker for the activation of cellular immunity. The physiological role of NP, in addition to the well-known importance of BH4 as an essential cofactor for nitric oxide synthase and aromatic amino acid hydroxylase, remains unclear. NP and BH4 are produced from guanosine triphosphate (GTP) [10,11] via several steps of an enzymatic reaction. GTP cyclohydrolase I (GCH) catalyzes the first and rate-limiting step, producing 7,8-dihydroneopterin triphosphate (NH2TP) (Figure 1). Research has suggested that GCH expression is induced by IFN-γ, which is released from activated Th1s and other cells [12], resulting in the elevation of GCH activity surpassing that of 6-pyruvoyl tetrahydropterin synthase (PTPS) in immune cells such as monocytes and macrophages, ultimately contributing to NP synthesis [13] (Figure 1).

Recently, NP has been reported to be a useful diagnostic marker for COVID-19 that correlates with disease severity and is a prognostic factor [14,15]. However, because sample collection time may affect the results, it is necessary to confirm the changes in NP over time from the onset of the disease; however, few reports have evaluated the changes in NP levels over time in patients with COVID-19. Furthermore, early predictive markers to distinguish between viral and bacterial infections are urgently needed, as secondary bacterial infections have been reported to complicate and cause severe disease in COVID-19 cases [7]. In animal experiments using mice with bacterial infection, increases in plasma BH4 and BH2 levels and organ damage have been observed [16]. These findings suggest an association between NP and the composite BP profile (BH4, BH2, and BP, collectively referred to as BPs) in the pathogenesis of bacterial infections. While some studies have explored the relationship between NP and viral infections [14,15] and a previous report investigated bacterial infections in connection with BPs [16], there is a shortage of studies directly comparing both infectious diseases despite their shared involvement in the same biosynthetic pathway. In this study, we investigated the relationship between the changes in plasma NP and BP levels over time and disease severity, as well as between plasma NP and BP levels in patients with COVID-19 and viral infections complicated by secondary bacterial infections to determine how different infectious agents affect the metabolites of the same biosynthetic pathway.

## 2. Materials and Methods

### 2.1. Objectives

#### 2.1.1. Patients with COVID-19

**Figure 2:**
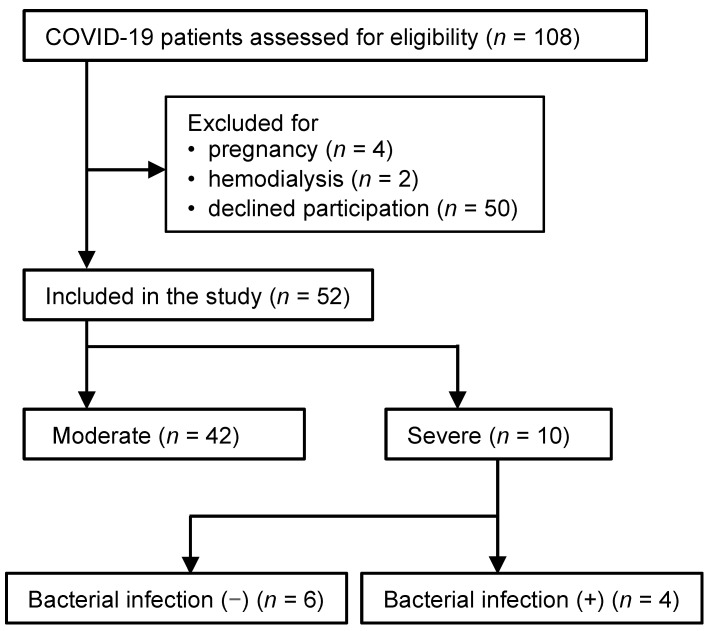
Flowchart of COVID-19 patients. Of 108 patients aged 18 years or older with positive SARS-CoV-2 polymerase chain reaction test results, 52 patients were included in the study, excluding pregnant patients, patients with chronic renal failure undergoing hemodialysis, patients who could not provide consent, and patients who were not enrolled. In addition, all 52 patients with COVID-19 were divided into a severe group (patients who received endotracheal intubation and mechanical ventilation during the study, *n* = 10) and a moderate group (patients who were not intubated and received oxygen, *n* = 42). Four of the ten patients in the severe group had complications due to secondary bacterial infections.

Of 108 patients over 18 years of age admitted to Kagoshima University Hospital and Kagoshima City Hospital between December 2020 and June 2021 with positive SARS-CoV-2 polymerase chain reaction test results, excluding those with pregnancy as well as those in chronic renal failure and undergoing hemodialysis, 52 patients were included. In addition, all 52 patients with COVID-19 were divided into a severe group (patients who received endotracheal intubation and mechanical ventilation during the study, *n* = 10) and a moderate group (patients who were not intubated and received oxygen, *n* = 42). Four of the ten patients in the severe group had secondary bacterial infections during treatment (Figure 2).

#### 2.1.2. Patients with Bacterial Sepsis (No Viral Infection)

A total of 25 patients aged 18 years or older admitted to the ICU of Kagoshima University Hospital between September and December 2017 and between January 2020 and May 2021, diagnosed with bacterial sepsis based on laboratory results and Sepsis-3 criteria, were included in the study. Patients with chronic renal failure undergoing hemodialysis and those without informed consent were excluded.

### 2.2. Experimental Design

#### Comparative Study of Various Parameters

A total of 52 patients with COVID-19 were divided into severe (*n* = 10) and moderate groups (*n* = 42), and laboratory values of various parameters, including plasma NP and plasma BPs on the day of admission, were compared. Next, to evaluate changes in measured parameters over time from the date of onset, we defined the date of onset as “any day on which cold symptoms, gastrointestinal symptoms, or taste/olfactory disorders begin to appear,” and changes in various parameters over time from the day of onset to day 14 were examined for the severe and moderate groups, divided into seven periods of 2 days each (periods I–VII). We also compared plasma NP and plasma BPs in patients with COVID-19 (*n* = 52) and bacterial sepsis (*n* = 25) to confirm differences in changes in plasma NP and plasma BPs due to viral and bacterial infections.

### 2.3. Laboratory Measurements

Lymphocyte (cells/μL) and neutrophil (cells/μL) counts were extracted from the electronic medical records of Kagoshima University Hospital and Kagoshima City Hospital. Plasma levels of interleukin-6 (IL-6) were assessed using ELISA (R&D Systems, Minneapolis, MN, USA), and plasma concentrations of IFN-γ were measured using ELISA kits (Proteintech, Rosemont, IL, USA). Plasma NP and BP levels were analyzed using high-performance liquid chromatography with fluorescence detection [17]. The institutional standard for SARS-CoV-2 requires the addition of perchloric acid to inactivate the virus in specimens. BH4, BH2, and BP levels were measured after oxidation of the samples from the people with COVID-19 with 1% I2 and 2% KI in 1N HCl for 60 min in the dark and at room temperature. Samples from patients with bacterial sepsis were treated with 0.2% dithioerythritol (final concentration), and BH4, BH2, and BP were measured separately using the post-column oxidation method [18]. The NP/BPs (N/B) ratio was calculated as an indicator of PTPS activity [19,20,21].

### 2.4. Sequential Organ Failure Assessment

Used worldwide for evaluating organ failure in intensive care settings, the SOFA score evaluates six items (respiration, coagulation, liver, cardiovascular, central nervous system, and renal), each of which is classified into five levels, from 0 to 4 [22].

### 2.5. Statistical Analysis

JMP^®^ Pro 17.1.0 (SAS Institute Inc., Cary, NC, USA) was used for statistical analysis. The Shapiro–Wilk test for normality was also employed. Continuous variables are expressed as medians (25th–75th percentile quartiles). The Wilcoxon test was used to compare continuous variables between the groups, and Spearman’s rank correlation coefficient rho was calculated for correlation analysis. Statistical significance was set at *p* < 0.05.

### 2.6. Ethics Approval and Consent to Participate

This two-center observational study was approved by the Ethics Committee on Epidemiological Studies of Kagoshima University and Kagoshima City Hospital (approval No. 200143epi, 230009epi). Informed consent was obtained from all patients.

## 3. Results

### 3.1. Background of Patients with COVID-19

The characteristics of the 52 patients with COVID-19 at the time of admission are shown in Table 1. There was no significant difference in the time from onset of symptoms to hospitalization between the severe and moderate groups of patients with COVID-19 (9 vs. 8 days). Plasma NP concentrations were higher than the reference range of 13.0 (11.5–15.0) pmol/mL in both the severe (125.8 [80.7–224.0] pmol/mL) and moderate groups (75.2 [47.9–112.6] pmol/mL). However, when comparing the two groups, the plasma NP in the severe group was significantly higher than that in the moderate group (*p* < 0.05). On the other hand, there was no significant difference in plasma BP concentration between the severe (13.4 [8.3–38.8] pmol/mL) and moderate groups (11.7 [9.0–16.6] pmol/mL), and the plasma BPs of both groups were within the standard range of 12.3 (10.6–14.1) pmol/mL. Regarding the other test parameters, compared to the moderate group, IL-6, CRP, and SOFA showed significantly higher values in the severe group, and IFN-γ and lymphocyte count showed significantly lower values in the severe group.

### 3.2. Changes in Measurement Parameters over Time

**Figure 3:**
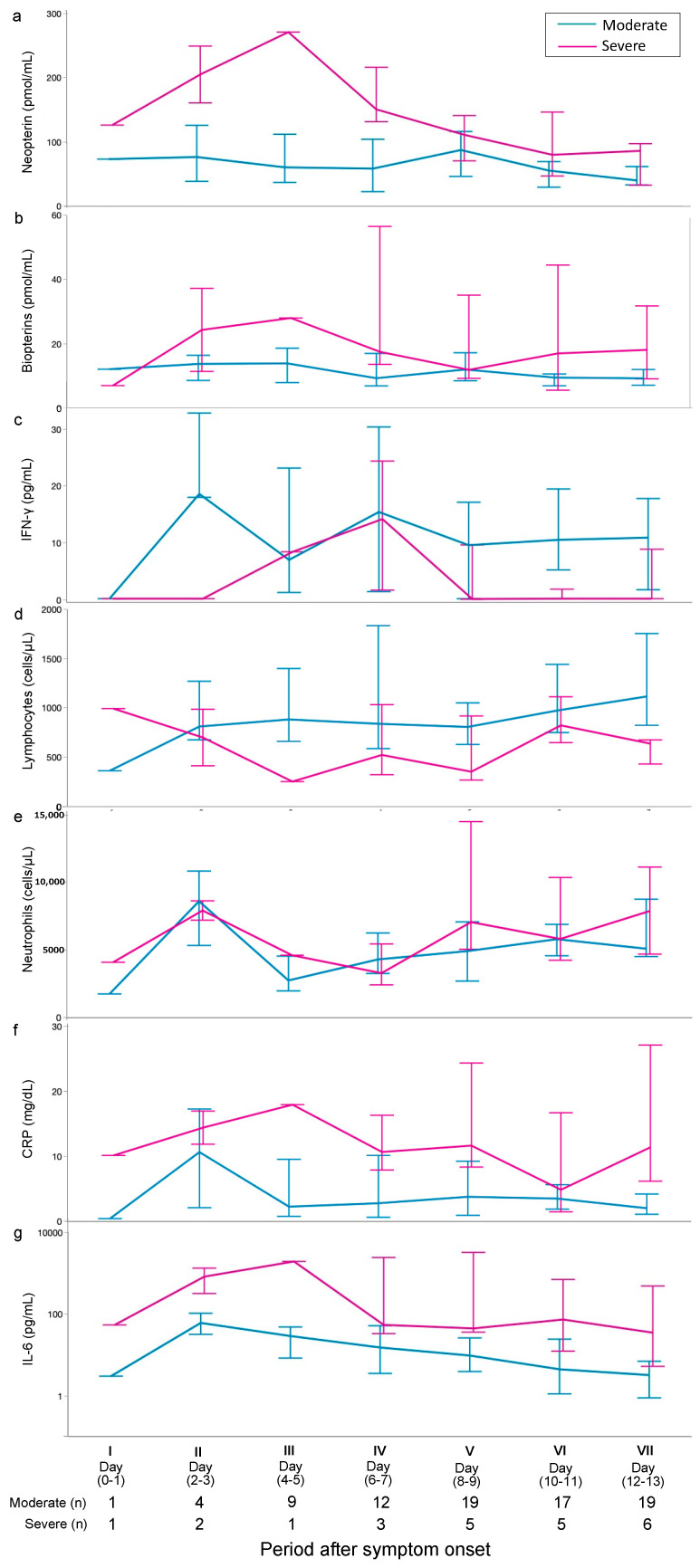
Changes over time in measured parameters in 52 COVID-19 patients. Plasma levels in the two groups were compared by period. Neutrophils (**e**) and IL-6 (**g**) are shown on a logarithmic scale. Comparing the severe and moderate disease groups, plasma NP (**a**) tended to be higher in the severe group than in the moderate group from period I to IV (days 0–7) but remained similar in both groups after period V (days 8–9). On the other hand, plasma BPs (**b**) tended to be higher in the severe group than in the moderate group throughout the entire measurement period. IL-6 and CRP (**f**) showed higher values in the severe group throughout the measurement period, but IFN-γ (**c**) and lymphocytes (**d**) showed lower values in the severe group. NP, neopterin; BPs, total biopterin; IFN-γ, interferon-γ; CRP, C-reactive protein; IL-6, interleukin-6.

Regarding changes over time from the day of onset, the plasma NP in both groups remained higher than the reference range of 13.0 (11.5–15.0) pmol/mL throughout the entire measurement period (days 0 to 14). When the severe and moderate disease groups were compared, the plasma NP tended to be higher in the severe group than in the moderate group from periods I to IV (days 0–7) but remained similar in both groups after period V (days 8–9). On the other hand, plasma BPs tended to be higher in the severe group than in the moderate group throughout the entire measurement period (days 0 to 14) but with values close to the reference range of 12.3 (10.6–14.1) pmol/mL. IL-6 and CRP showed higher values in the severe group throughout the measurement period (days 0 to 14), but IFN-γ and lymphocyte counts showed lower values in the severe group (Figure 3).

### 3.3. Time Course of Plasma NPs and BP Levels in Patients with Complicated Bacterial Infections

**Figure 4:**
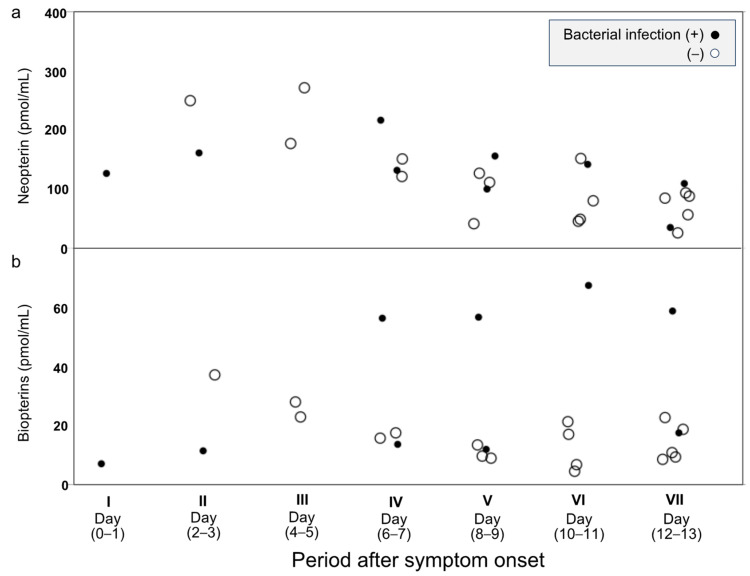
Time course of plasma NP (**a**) and BP (**b**) levels in patients with complicated bacterial infections. Four of the ten patients in the severe COVID-19 group experiences complications due to secondary bacterial infections. Compared to the plasma BPs of the 6 patients in the severe group who did not have bacterial infections, the plasma BPs of the 4 patients with secondary bacterial infections were higher, but plasma NP did not differ significantly between patients with and without bacterial infections.

Four of the 10 patients in the severe COVID-19 group were complicated by secondary bacterial infections (Figure 2, Table 2). Compared to the plasma BPs of the 6 patients in the severe group who did not have bacterial infections, the plasma BPs of the 4 patients with secondary bacterial infections were higher; however, plasma NPs did not differ significantly between patients with and without bacterial infections (Figure 4).

### 3.4. Plasma NP and BP Levels Due to Different Infectious Agents (COVID-19 vs. Bacterial Infection)

**Figure 5:**
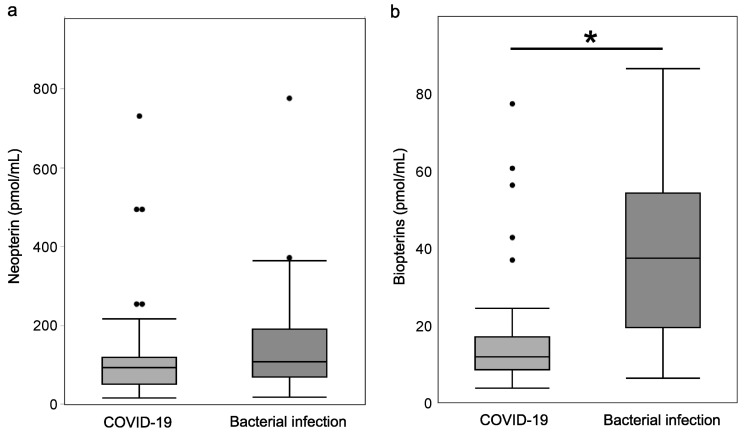
Plasma NP (**a**) and BP (**b**) levels due to different infectious agents (COVID-19 vs. bacterial infection). No significant difference in plasma NP was found, but plasma BPs were significantly higher in the bacterial sepsis group (37.6 [19.7–54.4] pmol/mL versus 12.2 [8.9–17.3] pmol/mL). * *p* < 0.05.

To see how the difference between viral and bacterial infections affected plasma NP and plasma BPs, we compared the bacterial sepsis group with the viral infection (COVID-19) group and found no significant difference in plasma NP. However, plasma BPs were significantly higher in the bacterial sepsis group (37.6 [19.7–54.4] pmol/mL versus 12.2 [8.9–17.3] pmol/mL, *p* < 0.05) (Figure 5).

## 4. Discussion

The findings of this study emphasize the need for the establishment of predictive markers for COVID-19. Furthermore, because the condition of some COVID-19 cases worsens when secondary bacterial infections occur, early predictive markers for the onset of bacterial infections are also needed. We examed the association of plasma NP and plasma BP levels over time with COVID-19 severity, and secondary bacterial infection. Plasma NP in patients with COVID-19 was approximately 10-fold higher in the severe group in this study and 6-fold higher in the moderate group compared to previously measured plasma NP levels in healthy subjects. As an indicator of cellular immune activation, NP is produced by monocytes and macrophages stimulated by IFN-γ [10]. Furthermore, it has been reported that in severe cases of COVID-19, IFN-γ is elevated in the acute phase due to high viral load and persistent inflammation [9]. In the present study, inflammatory markers such as CRP and IL-6 were elevated in the severe group, and SOFA values in the severe group showed that the patients were more severely ill than those in the moderate group. However, contrary to expectations, IFN-γ levels were lower in the severe group (Figure 3). The lack of IFN-γ elevation in the severe group could have resulted from the following factors: In severe coronavirus infections, the type I IFN response of infected cells is suppressed and the virus multiplies, leading to an overactive immune system response and a condition called the cytokine storm. When immune cells are overactivated, activated lymphocytes move from the circulation to tissues and act as a defense mechanism, but some activated lymphocytes are known to undergo apoptosis or cell death. In other words, it has been reported that in severe COVID-19 infection, the number of lymphocytes in the blood decreases, resulting in a decrease in the blood IFN-γ concentration [23]. Furthermore, it has been reported that when the amount of inhaled virus is high, the virus evasion mechanism may strongly suppress the IFN response, causing a delay in induction [24]. Although IFN-γ was low at admission in the severe COVID-19 group in the present study, the plasma NP was significantly elevated, suggesting a peak of IFN-γ that activated NP production before admission. In general, the incubation period for COVID-19 is approximately 5 days [25], and the period from onset of illness to hospitalization (first measurement) in the present severe group was approximately 9 days (Table 1). In other words, this IFN-γ level at the time of hospitalization was a test value approximately 14 days after infection with COVID-19. Based on the above, although IFN-γ was elevated in the early stages of infection (before hospitalization), the blood IFN-γ level must have already decreased approximately 14 days after infection (at the time of hospitalization) due to the viral evasion mechanism in severe COVID-19 infection and decreased lymphocyte count. Furthermore, plasma NP, which was significantly elevated at the onset of the disease, is also thought to have declined from day 7 of the disease, as the blood IFN-γ concentration decreased. Previous reports have suggested plasma NP as a useful marker for the diagnosis and severity of COVID-19 [14,15], but our study suggests that if plasma NP is to be used as an indicator of severity, the timing of blood collection is important, and the number of days since infection should be taken into account for evaluation.

The incidence of secondary bacterial infections in hospitalized patients with COVID-19 infection is reported to be 8.4%, and 39.9% in ICU patients [6], and, in this study, the mortality rate of patients with COVID-19 with complicated bacterial infections was 42.9% in the early bacterial infection group (<48 h after admission), 66% in the late bacterial infection group (2–14 days after admission), 48.1% for one bacterial infection, and 75.9% for two or more bacterial infections [7]. Furthermore, in a report comparing secondary infections after hospitalization for COVID-19 and influenza, the incidence of secondary infections was higher for COVID-19 than for influenza (12.6% vs. 8.7%), and the odds ratio of multiple infections for death was 1.12 for influenza but 7.64 for COVID-19, which was extremely high [6]. In summary, the early detection of secondary bacterial infections in COVID-19 is extremely important. While the prevalence of concurrent bacterial infections (infections diagnosed within 48 h after admission) in patients with COVID-19 is low at 3–8%, the rate of antibiotic use is extremely high at 50–75%. Additionally, among patients with COVID-19 infection accompanied by bacterial infection, the proportion of antibiotic-resistant infections has been reported to be 60.8% [6]. It has been suggested that the surge in COVID-19-related hospitalizations is associated with an increase in antibiotic-resistant infections, such as methicillin-resistant *Staphylococcus aureus* and vancomycin-resistant *enterococci* [26,27], and frequent use of antibiotics can worsen antimicrobial resistance. Therefore, antibiotic treatment should not be given as standard to hospitalized patients with COVID-19 infection unless bacterial infection is strongly suspected [6]. In other words, caution is needed regarding the easy prophylactic use of broad-spectrum antimicrobial agents for fear of secondary bacterial infections. For this reason, early predictive markers for the development of secondary bacterial infections in patients with COVID-19 infection are needed.

Regarding plasma BPs, all patients in the severe and moderate groups who did not develop secondary bacterial infections in this study were within the reference range, indicating that plasma BPs are not useful for diagnosing COVID-19 infection or determining its severity. In viral infections, the activation of GCH by IFN-γ stimulation from T cells and other cells [10,12] enhances NP synthesis but not PTPS activity, suggesting that the biosynthetic pathway to BPs is not activated. However, plasma BPs have been reported to be significantly elevated in animal models of bacterial sepsis [16] and in patients with bacterial septic shock [28]. In this study, plasma BPs were elevated in patients with COVID-19 infection who also had secondary bacterial infection, and plasma BPs in patients with bacterial sepsis were significantly higher than in patients with COVID-19 infection. Since PTPS activity and GCH activation are enhanced in bacterial sepsis due to the involvement of neutrophils and other factors, the biosynthetic pathway to BPs may be activated rather than NP, increasing plasma BPs. In other words, plasma BPs may be useful as an early predictive marker for the development of secondary bacterial infections in patients with COVID-19 infection. In summary, plasma NP and plasma BPs are potential markers for predicting viral and bacterial infections and may also be used as indicators to discriminate between viral and bacterial diseases.

Our study has several limitations. First, the sample size was small, with only 10 patients included in the severe disease group. Second, the small number of patients at disease onset may have prevented us from capturing the exact changes in each parameter. Finally, the incubation period of COVID-19 is approximately 5 days, and the median time between the onset of illness and the first measurement was approximately 8 days; it is possible that the IFN-γ levels in the blood were elevated during the incubation period of COVID-19, but the absence of symptoms precluded the measurement of IFN-γ levels in the blood during that time. Furthermore, the mechanism by which the biosynthetic pathways of NP and BPs are activated during infection may differ among pathogens, and further verification is needed for infections other than viral or bacterial (e.g., fungi). However, this study was significant because it confirmed that the plasma NP and plasma BPs in COVID-19 behave differently depending on the severity and secondary bacterial infection.

## 5. Conclusions

The increase in plasma NP in the early stage of onset (days 0 to 7) correlates with the severity of COVID-19 sepsis but does not reflect the severity thereafter; thus, the timing of specimen collection may affect severity assessment. Plasma BPs were found to increase when secondary bacterial infections occurred. This means that plasma NP and BPs may be useful markers for the diagnosis and severity of COVID-19 infection as well as for evaluating secondary bacterial infection complications. The biosynthetic pathways of NP and BPs may differ depending on pathogen type, and the activated mechanisms require further investigation.

## Figures and Tables

**Table 1:** Characteristics of the 52 patients with COVID-19 at time of admission.

		All Patients		Moderate		Severe		*p*-Value
		*n* = 52		*n* = 42		*n* = 10		
Sex	Male (%)	37	(71.2)	30	(69.77)	8	(72.73)	
	Female (%)	15	(28.8)	13	(30.23)	3	(27.27)	
Age (IQR)		63	(53–71)	61	(52–68)	69	(66–77)	<0.05
From onset of symptom to hospital admission, median (IQR) (days)		8	(5–10)	8	(5–10)	9	(5.3–11.3)	NS
SOFA score (IQR)		2	(1–4)	2	(1–3)	5	(3–8)	<0.05
Hospital mortality (%)		9.8		0		36.4		
Laboratory findings, median (IQR)								
Neopterin (pmol/mL)		93.0	(49.7–117.9)	75.2	(47.9–112.6)	125.8	(80.7–224.0)	<0.05
Biopterin (pmol/mL)		12.2	(8.9–17.3)	11.7	(9.0–16.6)	13.4	(8.3–38.8)	NS
Neopterin/biopterin ratio		6.3	(5.1–8.1)	6.2	(5.2–7.4)	9.0	(4.5–12.4)	NS
IFN-γ (pg/mL)		7.0	(0.2–14.8)	9.3	(0.2–17.4)	0.2	(0.2–6.4)	<0.05
Lymphocyte (cells/μL)		740	(472.3–1032)	804	(572–1084)	447	(298–817)	<0.05
Neutrophil (cells/μL)		4360	(2673–7061)	4391	(2508–6090)	4237	(3213–11,807)	NS
Neutrophil/lymphocyte ratio		6.1	(3.1–10.6)	5.8	(3.0–9.3)	11.9	(4.5–35.6)	<0.05
CRP (mg/dL)		5.6	(1.9–10.4)	4.5	(1.5–9.5)	10.3	(7.2–15.1)	<0.05
IL-6 (pg/mL)		24.9	(2.8–46.1)	18.3	(1.3–36.9)	49.0	(24.7–372.0)	<0.05

NS: not significant.

**Table 2:** Bacterial culture results for patients with COVID-19 with bacterial infection.

#	Sex	Age	Source	Organisms
1	F	88	Blood	*Staphylococcus aureus*, *Haemophilus influenzae*, *Corynebacterium species*
2	M	66	Lung	*Staphylococcus aureus*, *Streptococcus pneumoniae*, *Haemophilus influenzae*
3	F	76	Blood	*Escherichia coli*
4	M	66	Blood	*Escherichia coli*

Abbreviations: F, female; M, male.

## Data Availability

The data that support the findings of this study are available from the corresponding author upon reasonable request.
